# Supplementary material for: Causal Relationship between Aging and Anorexia Nervosa: A White-Matter-Microstructure-Mediated Mendelian Randomization Analysis
Source: Biomedicines. 2024 Aug 16;12(8):1874. doi: 10.3390/biomedicines12081874 (PMC11351342; doi:10.3390/biomedicines12081874)
Supplement: Supplementary file 1 [file biomedicines-12-01874-s001.zip › Supplementary Figure/Figure S4/Singe SNP plot of MR analysis for TL to ubm-b-2046.pdf]

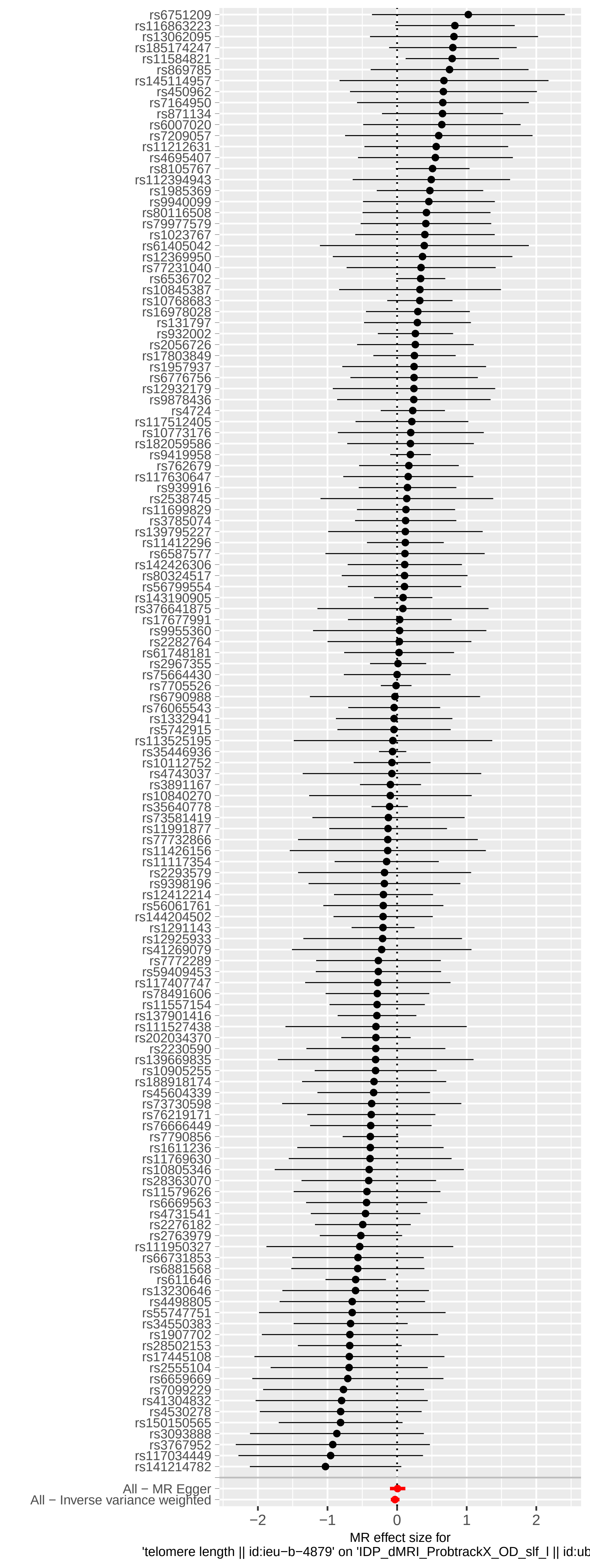
rs6751209
rs116863223
rs13062095
rs185174247
rs11584821
rs869785
rs145114957
rs450962
rs7164950
rs871134
rs6007020
rs7209057
rs11212631
rs4695407
rs8105767
rs112394943
rs1985369
rs9940099
rs80116508
rs79977579
rs1023767
rs61405042
rs12369950
rs77231040
rs6536702
rs10845387
rs10768683
rs16978028
rs131797
rs932002
rs2056726
rs17803849
rs1957937
rs6776756
rs12932179
rs9878436
rs4724
rs117512405
rs10773176
rs182059586
rs9419958
rs762679
rs117630647
rs939916
rs2538745
rs11699829
rs3785074
rs139795227
rs11412296
rs6587577
rs142426306
rs80324517
rs56799554
rs143190905
rs376641875
rs17677991
rs9955360
rs2282764
rs61748181
rs2967355
rs75664430
rs7705526
rs6790988
rs76065543
rs1332941
rs5742915
rs113525195
rs35446936
rs10112752
rs4743037
rs3891167
rs10840270
rs35640778
rs73581419
rs11991877
rs77732866
rs11426156
rs11117354
rs2293579
rs9398196
rs12412214
rs56061761
rs144204502
rs1291143
rs12925933
rs41269079
rs7772289
rs59409453
rs117407747
rs78491606
rs11557154
rs137901416
rs111527438
rs202034370
rs2230590
rs139669835
rs10905255
rs188918174
rs45604339
rs73730598
rs76219171
rs76666449
rs7790856
rs1611236
rs11769630
rs10805346
rs28363070
rs11579626
rs6669563
rs4731541
rs2276182
rs2763979
rs111950327
rs66731853
rs6881568
rs611646
rs13230646
rs4498805
rs55747751
rs34550383
rs1907702
rs28502153
rs17445108
rs2555104
rs6659669
rs7099229
rs41304832
rs4530278
rs150150565
rs3093888
rs3767952
rs117034449
rs141214782
All − MR Egger
All − Inverse variance weighted
−2
−1
0
1
2
MR effect size for
'telomere length || id:ieu−b−4879' on 'IDP_dMRI_ProbtrackX_OD_slf_l || id:ub
